# Killing Two Birds With One Stone: Effective Control of Both Non-Small Cell Lung Cancer and Progressive Multifocal Leukoencephalopathy With Atezolizumab, A Case Report

**DOI:** 10.3389/fimmu.2022.889148

**Published:** 2022-05-03

**Authors:** Nicolas Lambert, Majdouline El Moussaoui, Caroline Ritacco, Martin Moïse, Astrid Paulus, Philippe Delvenne, Frédéric Baron, Bernard Sadzot, Pierre Maquet

**Affiliations:** ^1^Department of Neurology, University Hospital of Liège, Liège, Belgium; ^2^Laboratory of Molecular Regulation of Neurogenesis, GIGA-Stem Cells and GIGA-Neurosciences, Interdisciplinary Cluster for Applied Genoproteomics (GIGA-R), University of Liège, Liège, Belgium; ^3^Department of Infectious Diseases and General Internal Medicine, University Hospital of Liège, Liège, Belgium; ^4^Hematology Research Unit, GIGA-I3, Interdisciplinary Cluster for Applied Genoproteomics (GIGA-R), University of Liège, Liège, Belgium; ^5^Department of Radiology, University Hospital of Liège, Liège, Belgium; ^6^Department of Pneumology, University Hospital of Liège, Liège, Belgium; ^7^Department of Anatomopathology, University Hospital of Liège, Liège, Belgium; ^8^Department of Hematology, University Hospital of Liège, Liège, Belgium

**Keywords:** PML - progressive multifocal leucoencephalopathy, JC virus (JCV), immune checkpoint inhibitors (ICI), immunotherapy, atezolizumab, PD1 and PDL1, non small cell lung cancer (NSCLC), lung adecarcinoma

## Abstract

Treating patients with cancer complicated by severe opportunistic infections is particularly challenging since classical cancer treatments, such as chemotherapy, often induce profound immune suppression and, as a result, may favor infection progression. Little is known about the potential place of immune checkpoint inhibitors in these complex situations. Here, we report a 66-year-old man who was concomitantly diagnosed with non-small cell lung cancer and progressive multifocal leukoencephalopathy. The patient was treated with anti-PD-L1 antibody atezolizumab, which allowed effective control of both lung cancer and progressive multifocal leukoencephalopathy, as demonstrated by the patient’s remarkable neurologic clinical improvement, JC viral load reduction in his cerebrospinal fluid, regression of the brain lesions visualized through MRI, and the strict radiological stability of his cancer. In parallel, treatment with atezolizumab was associated with biological evidence of T-cell reinvigoration. Hence, our data suggest that immune checkpoint inhibitors may constitute a treatment option for patients with cancer complicated by severe opportunistic infections.

## Introduction

Over the past decade, immune checkpoint inhibitors (ICIs) promoted dramatic advances in cancer treatment. These innovative therapies enable durable clinical response for a subset of patients with unresectable malignancies, including advanced non-small cell lung cancer (NSCLC), which is the largest single contributor of cancer deaths in the United States (1). More recently, ICIs targeting programmed cell death 1 (PD1) or its ligand (PD-L1) were proposed for the treatment of progressive multifocal leukoencephalopathy (PML) (2, 3). The latter is a devastating brain infectious disease caused by JC virus (JCV) in the course of cellular immune deficiency. Since there is no effective anti-viral treatment for PML, survival depends on the ability to achieve timely immune reconstitution. Otherwise, the prognosis is particularly grim with mortality rate reaching 90% for hematologic malignancies-associated PML (4). We hereby report a patient who was concomitantly diagnosed with NSCLC and PML and successfully treated for both with atezolizumab monotherapy.

## Case Report

**Figure 1:**
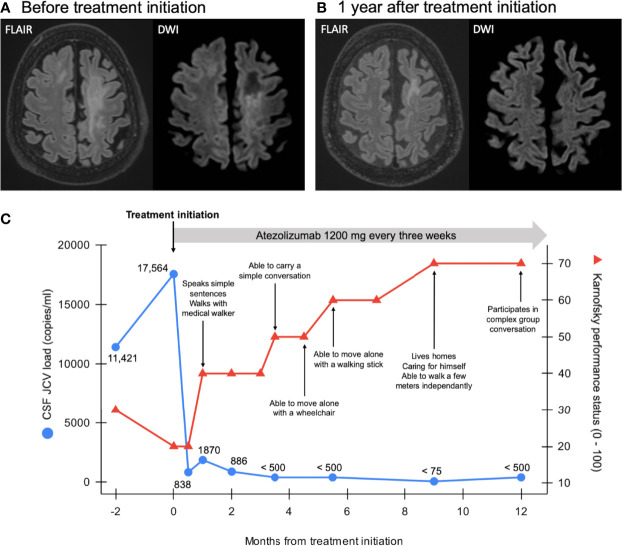
Panel **(A)** Brain MRI scan at admission showing T2-fluid attenuated inversion recovery (FLAIR)-weighted multifocal hyperintense white matter lesions with diffusion-weighted imaging (DWI, narrowed windowing for clarity purpose) ill-defined hyperintense edges and hypointense core in keeping with disease activity. Panel **(B)** Brain MRI scan 1 year after treatment initiation showing regression of the T2/FLAIR hyperintense lesions and disappearance of the DWI (narrowed windowing) hypersignal, in keeping with gliosis (ADC not shown). Note progressive brain atrophy in previously affected areas. Panel **(C)** Patient’s clinical course and evolution of JC viral load in the cerebrospinal fluid (CSF) over time. JC virus PCR assay’s quantification limit is 500 copies/ml; detection limit is 75 copies/ml.

A 66-year-old Caucasian man was admitted for severe aphasia, dysphagia, right hemiparesis, and gait impairment progressing for 1 month, making the patient bedridden and unable to communicate in any way (see **Supplementary Video**). Karnofsky performance status was evaluated at 30 (severely disabled). His medical history mainly consisted of former smoking, chronic alcohol consumption, gouty arthritis, arterial hypertension, and bioprosthetic aortic valve replacement. Brain magnetic resonance imaging (MRI) showed T2-weighted-fluid-attenuated inversion recovery (T2-FLAIR) hyperintense bilateral multifocal non-hemorrhagic white matter lesions without any mass effect nor enhancement after intravenous gadolinium injection. On diffusion-weighted imaging (DWI), these lesions exhibited typical ill-defined hyperintense edging and hypointense core suggestive of an active-stage disease (**Figure 1A** and **Supplementary Figure S1**). Cerebrospinal fluid (CSF) analysis revealed normal white blood cell count and protein level. Polymerase chain reaction (PCR) assay found 11,421 JCV copies/ml of CSF, upholding a definite diagnosis of PML defined by the American Academy of Neurology diagnostic criteria (5).

**Figure 2:**
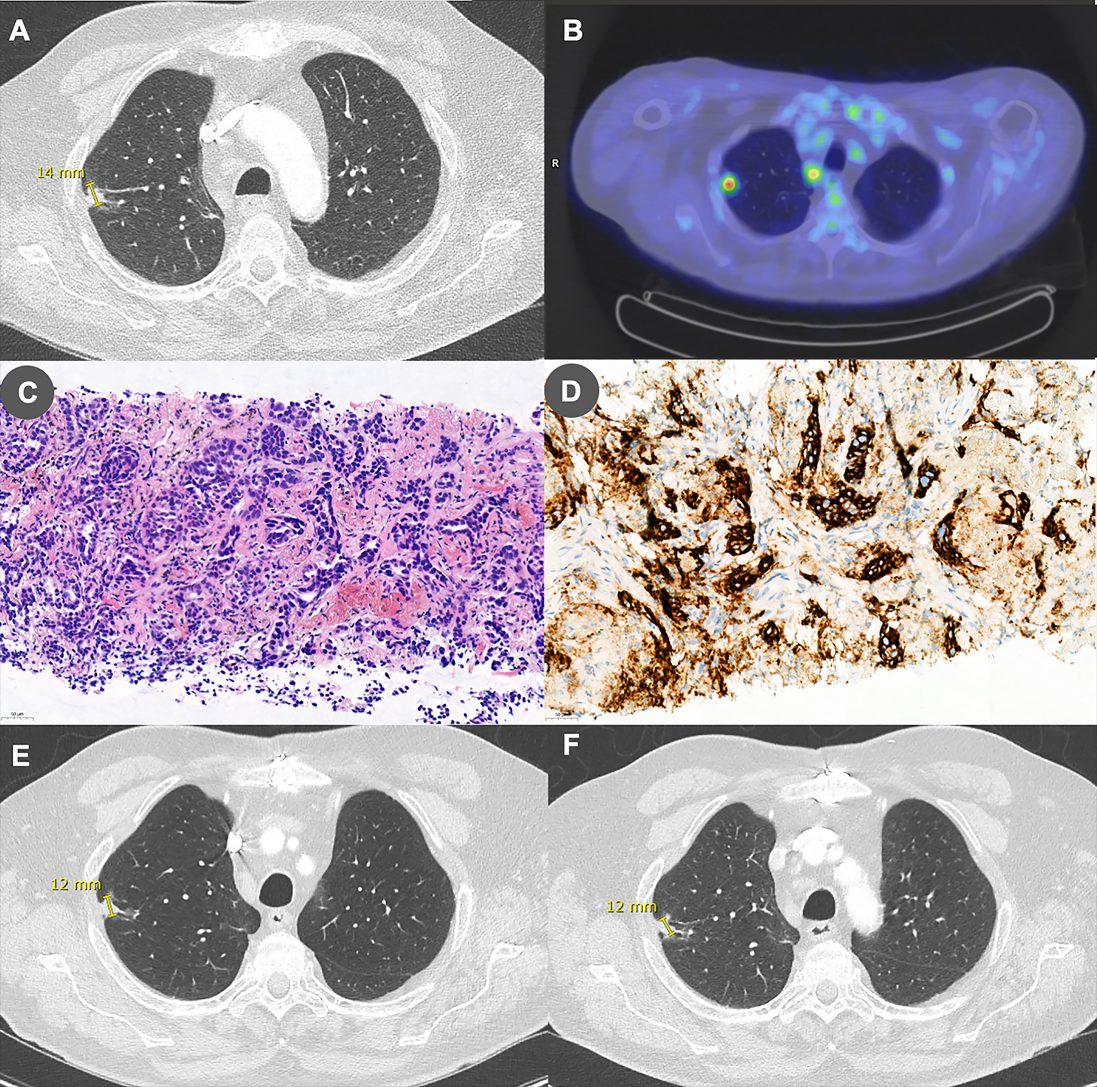
Panel **(A)** Chest CT performed before treatment initiation showing a 14-mm right upper lobe irregular nodular lesion. Panel **(B)** This lesion was found to be hypermetabolic on [^18^F]-fluorodeoxyglucose positron emission tomography. Panel **(C)** Lung biopsy specimen showing a modified fibrous tissue infiltrated by irregular and ramified tubuloacinar structures formed by non-small neoplastic cells with hyperchromatic nucleus and weakly eosinophilic cytoplasm. The overall appearance is compatible with a lung adenocarcinoma (hematoxylin-eosin stain, 200x). Panel **(D)** PD-L1 is expressed by 50% of tumor cells (PD-L1 immunoperoxydase, 200x). Panels **(E, F)** Chest CT performed **(E)** 6 months and **(F)** 1 year after treatment initiation showing stability of the lesion.

Complete blood count and flow cytometry were performed to search an underlying immune deficiency and revealed lymphopenia affecting predominantly CD4+ T cells (530 lymphocytes/μL including 140 CD4+ cells/μL, 210 CD8+ cells/μL, and 100 CD19+ cells/μL). Human immunodeficiency virus (HIV) serology was repeatedly negative on two separate samples. Whole-body [^18^F]-fluorodeoxyglucose positron emission tomography (^18^FDG-PET) and associated chest computed tomography (CT) revealed a 1.4 cm hypermetabolic nodular and irregular mass located at the lateral edge of the right upper lobe associated with ipsilateral hilar, paratracheal and subcarinal hypermetabolic lymphadenopathy (**Figures 2A, B**). Lung CT-guided biopsy was performed, and pathological analyses were consistent with the diagnosis of lung adenocarcinoma (**Figure 2C**). PD-L1 was expressed by 50% of tumor cells (**Figure 2D**). Anaplastic lymphoma kinase (ALK) and ROS1 immunohistochemical analyses were negative. We found no mutation in *EGFR* gene and next-generation sequencing (NGS) assay only found a *MET* mutation with no clinical impact. The patient was eventually diagnosed with PML in a context of stage IIIA (T1bN2M0) NSCLC according to the eighth American joint committee on cancer classification.

To reinvigorate both anti-JCV and anti-tumor immunity, the patient was started on atezolizumab, an anti-PD-L1 humanized monoclonal antibody, at a dosage of 1200 mg every 3 weeks. Clinical follow-up consisted of monthly physical and neurological examinations. Radiological follow-up consisted of brain MRI and thoracic-abdominal-pelvic CT every 3 months. JCV viral load in the CSF was evaluated by PCR assay at least every 3 months. To monitor immune exhaustion, we performed immunophenotyping using multicolor flow cytometry on peripheral blood mononuclear cells (PBMC) isolates collected the day before and 5 weeks after treatment initiation as well as on PBMC isolates from a healthy control subject.

One month after treatment initiation, the patient started to improve clinically. After 6 weeks, he was able to speak simple sentences and to walk in the corridor with a walker and the physiotherapist’s aid. CSF JCV load was considerably reduced to 1870 copies/ml (**Figure 1C**) and brain MRI was stable, without any enhancement after gadolinium injection. In parallel, detection of PD1 on the patient’s peripheral CD8+ and CD4+ T cells, which was noticeably high while compared to the healthy control subject, as well as detection of T cell immunoreceptor with Ig and ITIM domain (TIGIT), another inhibitory immune checkpoint, decreased with atezolizumab treatment (**Supplementary Figure S2**). As expected, PD-L1 expression drastically decreased on monocytes. No substantial change in CD3+, CD4+, and CD8+ cell counts was observed after treatment. Over the same period, the patient experienced two episodes of mild right wrist and ankle oligoarthritis which were successfully treated with non-steroidal anti-inflammatory drugs and colchicine. After the second event, colchicine was pursued at a daily dose of 0.5 mg and arthritis never recurred.

The patient was discharged to a revalidation center, where he continued to improve gradually. CSF JCV load reduced accordingly and was even below the PCR’s detection limit (75 copies/mL) 9 months after treatment initiation (**Figure 1C**). Brain MRI showed regression of the extent of the T2-FLAIR lesions and disappearance of DWI signs of disease activity (**Figure 1B** and **Supplementary Figure S1**). Regarding lung adenocarcinoma, repeated thoracic-abdominal-pelvic CTs demonstrated a stable disease (**Figures 2E, F**).

At the time of the most recent follow-up visit, over 1 year after treatment initiation, the patient lives independently at home and is still improving. He can walk at least 100 m without assistance, eat with no restriction, and participate in complex group conversations although some degree of aphasia remains (see **Supplementary Video**). He does not report any respiratory nor other cancer-related symptoms and adenocarcinoma has remained perfectly radiologically stable. Karnofsky performance status is evaluated at 70 (able to care for self).

## Discussion

**Figure 3:**
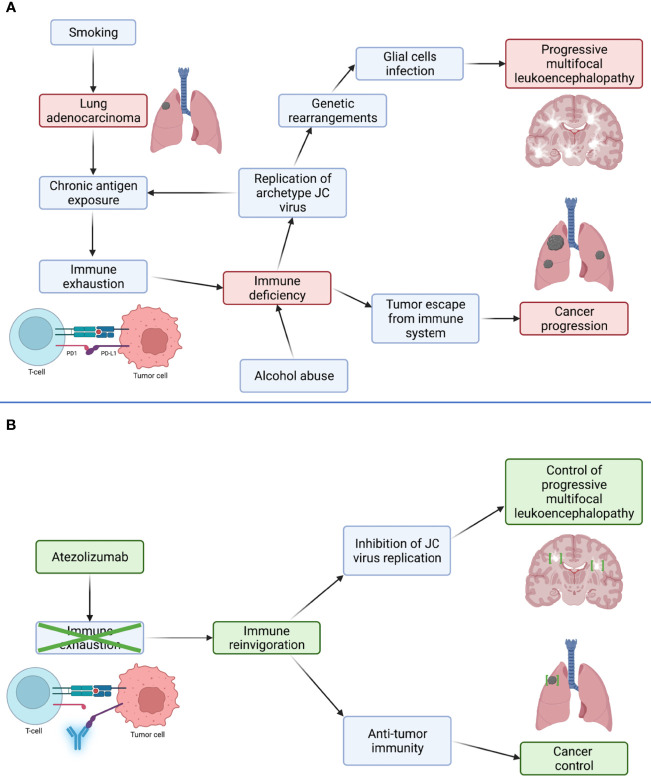
Proposed mechanism through which control of lung adenocarcinoma and progressive multifocal leukoencephalopathy (PML) was obtained with atezolizumab. Panel **(A)** In basal condition, adenocarcinoma induces immune exhaustion, a phenomenon mediated by inhibitory immune checkpoints, such as PD1, and characterized by progressive loss of immune cells effector functions. Resulting immune deficiency allows tumor escape from the immune system leading to its progression, but also replication of archetype JC virus (JCV). JCV accumulates replication-driven genetic rearrangements, conferring it the ability to infect glial cells and cause PML. Panel **(B)** Anti-PD-L1 antibody atezolizumab disrupts the interaction between PD1 and its ligand, PD-L1, mediating immune exhaustion. This disruption leads to immune cells reinvigoration, permitting the immune system to regain control of both lung adenocarcinoma and JCV replication and, therefore, control of PML.

Primary infection with archetype JCV, the transmissible and non-neurotropic form of the virus, typically occurs during childhood and leads to lifelong asymptomatic infection in immunocompetent hosts. However, in the course of cellular immune deficiency, JCV reactivation may occur, leading to replication-driven genetic rearrangements, thereby conferring the virus the ability to cause lytic oligodendrocytes infection and, therefore, PML (**Figure 3A**) (4, 6). Nowadays, HIV infection, lymphoproliferative disorders, and immunosuppressive or immunomodulatory drugs account for most PML cases (4). Less frequently, PML may occur during the course of solid cancers, even in the absence of any immunosuppressive therapy. Indeed, cancers, as chronic infections, induce immune exhaustion, a state of functional adaptation of immune cells mediated by inhibitory immune checkpoints and notably characterized by loss of effector functions in response to chronic antigen exposure (7). There is an increasing body of evidence that immune exhaustion, and notably the PD1/PD-L1 pathway, is involved in PML pathophysiology (2). Although we acknowledge the limitations of single-case reporting, we postulate that atezolizumab successfully counteracted immune exhaustion in our patient, which reinvigorated anti-tumor and anti-JCV immunity, as suggested by the patient’s clinical, virological, and radiological improvement as well as by the reduction of PD1 and TIGIT expression on peripheral blood T cells, allowing effective control of both diseases (**Figure 3B**).

Over the past three years, several reports suggested that blocking the PD1/PD-L1 pathway might be the long-awaited treatment for PML (2). However, these initial enthusiastic results have been tempered by later reports depicting sustained neurological degradation and death despite anti-PD1 therapy, thereby highlighting the need to determine patients most susceptible to benefit from this treatment (8). Based on the few observations reported thus far, several risk factors for treatment failure have been suggested, such as high CSF JCV copy number, concomitant use of immunosuppressive drugs, absence of pre-treatment anti-JCV activity detected *in vitro*, and more terminally exhausted phenotypes of immune cells (6, 8, 9). However, a robust and reproductible strategy to determine treatment-responsive patients is yet to be determined and should be investigated by further studies. Another caveat when using PD1/PD-L1 pathway inhibitors to treat PML is the risk of developing immune-related adverse events (irAEs) or immune-reconstitution inflammatory syndrome (IRIS), which is defined by a clinical deterioration temporally associated with immune reinvigoration. A close clinical follow-up is therefore essential when a patient is started on this therapeutic class to detect adverse events promptly. Since treatment of these usually relies on corticosteroids, which impairs anti-JCV immunity, benefits expected from treating IRIS and irAEs must be balanced with the risk of favoring PML progression (3). To date, the optimal management for these complex clinical situations is still unknown and should be further investigated.

Treating patients with cancer complicated by potentially deadly opportunistic infections is particularly challenging since classical oncologic treatments, such as chemotherapy, often induce even more profound immune deficiency, thereby favoring infection progression. Our case suggests that ICIs constitute a reasonable treatment option for a subset of these particularly complex situations since immune checkpoint blockade could be effective for treating both the infectious disease and the underlying cancer. Hence, beyond PML, ICIs have been used successfully to achieve control of other severe opportunistic infections, such as mucormycosis or aspergillosis (10, 11). Moreover, anti-PD1 treatment nivolumab reportedly induced complete remission of an HPV-positive head and neck squamous cell carcinoma in a patient with a previous history of PML without inducing infection relapse nor neurological immune-related adverse events (12).

## Data Availability Statement

The original contributions presented in the study are included in the article/**Supplementary Material**. Further inquiries can be directed to the corresponding author.

## Ethics Statement

Ethical review and approval was not required for the study on human participants in accordance with the local legislation and institutional requirements. The patients/participants provided their written informed consent to participate in this study. Written informed consent was obtained from the individual(s) for the publication of any potentially identifiable images or data included in this article.

## Author Contributions

NL conceived the idea of the study and drafted the manuscript. CR and FB performed flow cytometry experiments and analyses. Each author participated in the patient’s medical care and revised the manuscript for scientific content. All authors contributed to the article and approved the submitted version.

## Funding

NL and ME and MM are PhD students of the F.R.S.-F.N.R.S. Belgium. CR is a PhD student Télévie. FB and PM are respectively senior research associate and research director of the F.R.S.-F.N.R.S. Belgium.

## Conflict of Interest

The authors declare that the research was conducted in the absence of any commercial or financial relationships that could be construed as a potential conflict of interest.

## Publisher’s Note

All claims expressed in this article are solely those of the authors and do not necessarily represent those of their affiliated organizations, or those of the publisher, the editors and the reviewers. Any product that may be evaluated in this article, or claim that may be made by its manufacturer, is not guaranteed or endorsed by the publisher.

## Supplementary Material

Supplementary Figure 1 Brain MRI over the course of 12 months showed a reduction of the extent of both the T2-FLAIR lesions and the edge of restricted diffusion. There was no enhancement after gadolinium injection to suggest immune reconstitution inflammatory syndrome. ADC, apparent diffusion coefficient; FLAIR; fluid attenuated inversion recovery; DWI, diffusion-weighted imaging; T1 + G, T1-weighted sequences after gadolinium injection.

Supplementary Figure 2 Multicolor flow cytometry performed on patient’s peripheral blood mononuclear cell (PBMC) isolates collected the day before and 5 weeks after treatment initiation as well as on PBMC isolates from a healthy control subject. Panel **(A)** Reduction of PD1 expression on peripheral CD8+ and CD4+ T cells following atezolizumab initiation. Panel **(B)** Reduction of PD-L1 expression on peripheral monocytes following atezolizumab initiation. PD-L1 was already weakly expressed by B cells and T cells before treatment. Panel **(C)** Reduction of TIGIT expression on peripheral CD8+ and CD4+ T cells following atezolizumab initiation. Methods: Frozen PBMCs from the patient collected the day before and 5 weeks after atezolizumab initiation as well as from a healthy control subject were thawed and washed twice in a staining buffer (DPBS+ 3% FBS). PBMCs were then counted on ABX Micros 60 analyzer and 2 x106 PBMCs were engaged in the staining. These were stained 20 min in the dark at 4°C with the following anti-human antibodies: CD3-V450 (UCHT1), CD4-BV786 (SK3), CD8-PE (HIT8a), CD19-BUV395 (3G8), CD56-APC R700 (NCAM16.2), CD19-PECy5 (HIB19), CD14-APC (M5E2), PD1-BV650 (EH12.2H7), PD1L-PECy7 (MIH1), and TIGIT- PE/Dazzle594 (A15153G). PBMCs were washed twice with the staining buffer and stained 30 min in the dark at 4°C with fixable viability dye 520. Finally, PBMCs were washed twice in the same staining buffer and analyzed on FACS BD LSR Fortessa. Red: One day before atezolizumab initiation. Blue: Five weeks after atezolizumab initiation. Green: Healthy control subject. FMO, fluorescence minus one.

Supplementary Video Evolution of the patient’s clinical status over time. At admission, patient’s neurologic clinical evaluation was notably relevant for severe aphasia, astasia-abasia, and right hemiparesis. One year after treatment initiation, his clinical examination showed improvement of aphasia and complete resolution of the right hemiparesis. The patient is now able to get up and walk independently.
