# Supplementary figures and images for: Killing Two Birds With One Stone: Effective Control of Both Non-Small Cell Lung Cancer and Progressive Multifocal Leukoencephalopathy With Atezolizumab, A Case Report
Source: Front Immunol. 2022 May 3;13:889148. doi: 10.3389/fimmu.2022.889148 (PMC9110816; doi:10.3389/fimmu.2022.889148)

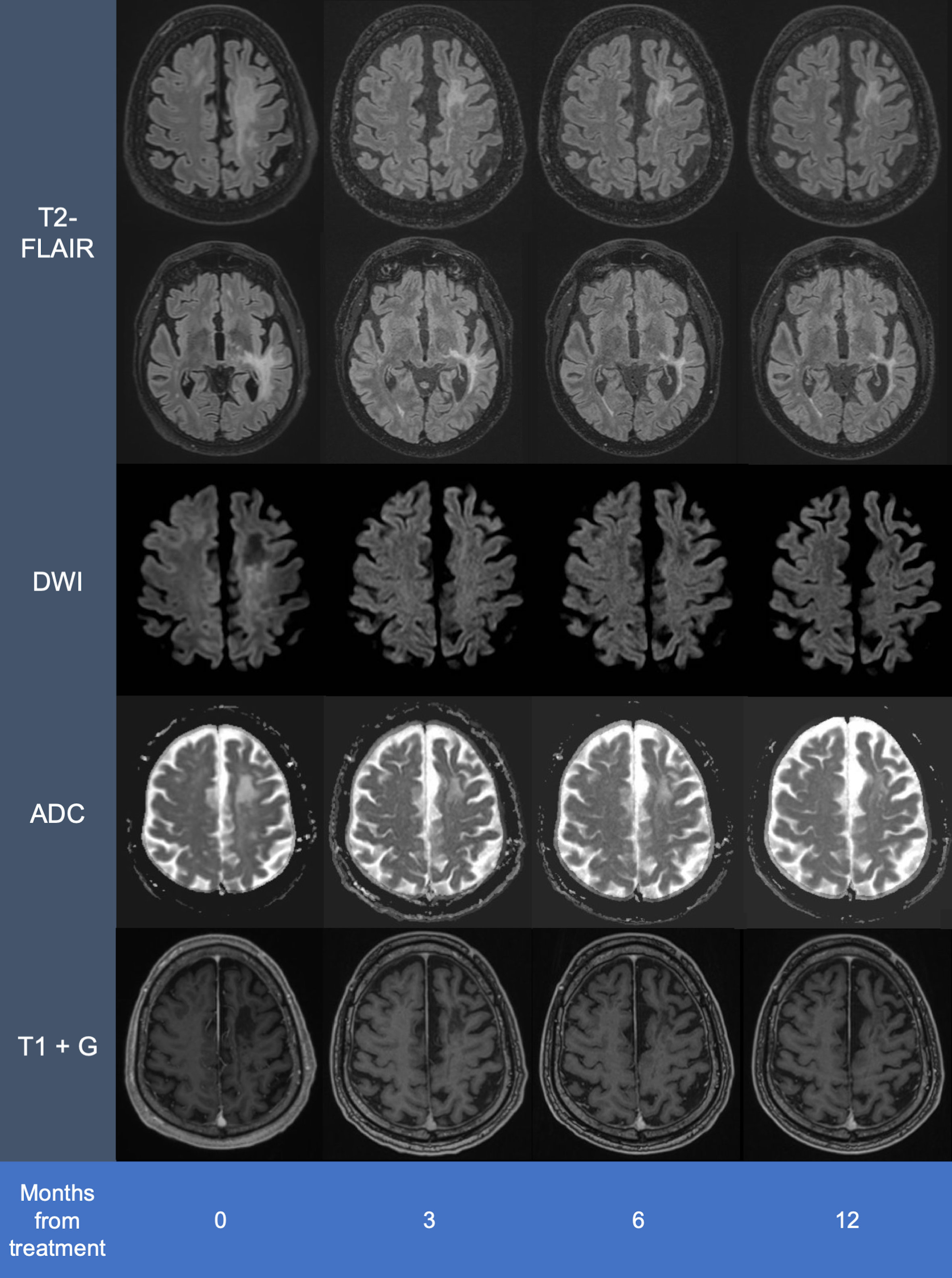

Supplement: Supplementary Figure 1 — Brain MRI over the course of 12 months showed a reduction of the extent of both the T2-FLAIR lesions and the edge of restricted diffusion. There was no enhancement after gadolinium injection to suggest immune reconstitution inflammatory syndrome. ADC, apparent diffusion coefficient; FLAIR; fluid attenuated inversion recovery; DWI, diffusion-weighted imaging; T1 + G, T1-weighted sequences after gadolinium injection. [file Image_1.tiff]

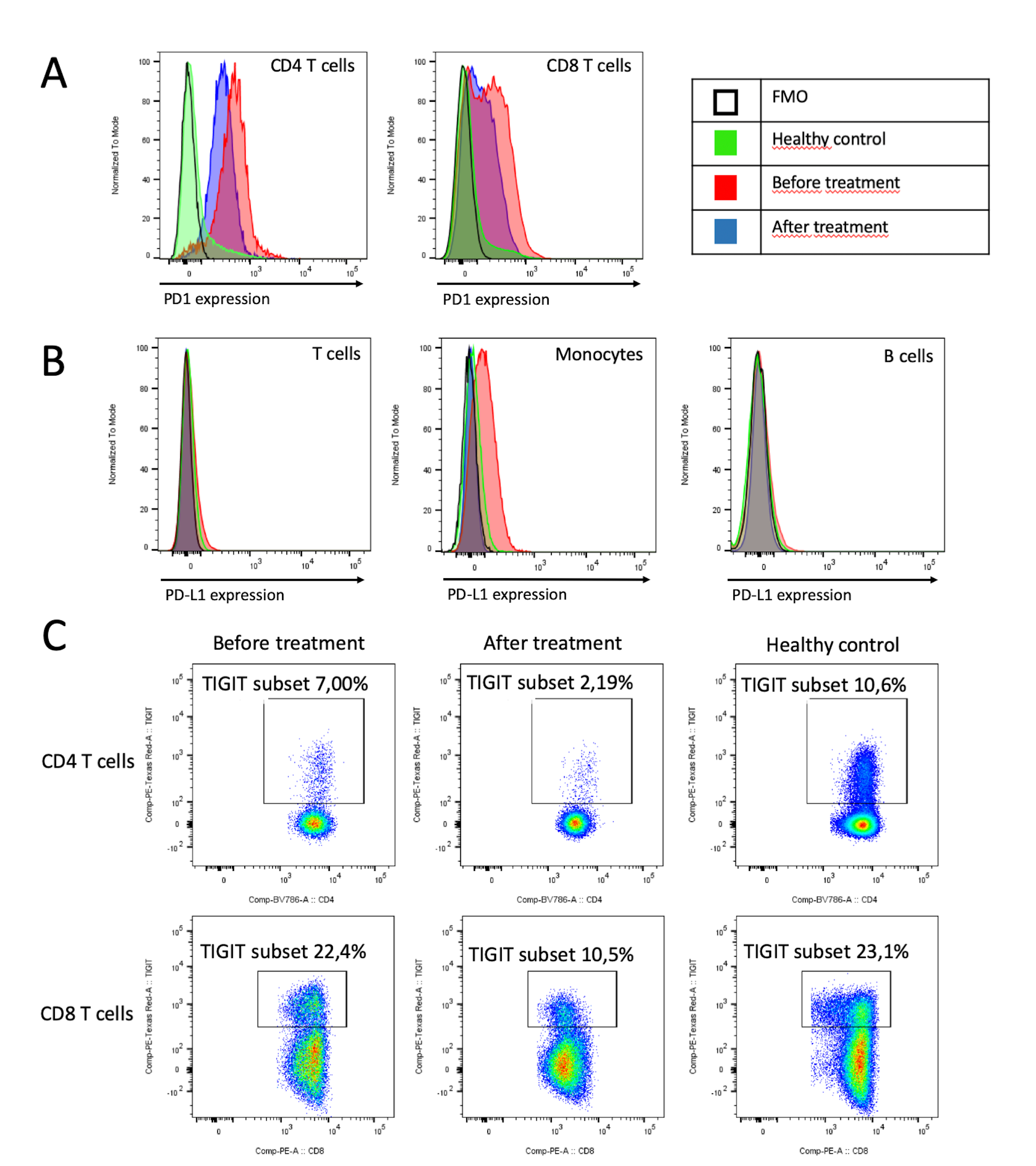

Supplement: Supplementary Figure 2 — Multicolor flow cytometry performed on patient’s peripheral blood mononuclear cell (PBMC) isolates collected the day before and 5 weeks after treatment initiation as well as on PBMC isolates from a healthy control subject. Panel (A) Reduction of PD1 expression on peripheral CD8+ and CD4+ T cells following atezolizumab initiation. Panel (B) Reduction of PD-L1 expression on peripheral monocytes following atezolizumab initiation. PD-L1 was already weakly expressed by B cells and T cells before treatment. Panel (C) Reduction of TIGIT expression on peripheral CD8+ and CD4+ T cells following atezolizumab initiation. Methods: Frozen PBMCs from the patient collected the day before and 5 weeks after atezolizumab initiation as well as from a healthy control subject were thawed and washed twice in a staining buffer (DPBS+ 3% FBS). PBMCs were then counted on ABX Micros 60 analyzer and 2 x10^6^ PBMCs were engaged in the staining. These were stained 20 min in the dark at 4°C with the following anti-human antibodies: CD3-V450 (UCHT1), CD4-BV786 (SK3), CD8-PE (HIT8a), CD19-BUV395 (3G8), CD56-APC R700 (NCAM16.2), CD19-PECy5 (HIB19), CD14-APC (M5E2), PD1-BV650 (EH12.2H7), PD1L-PECy7 (MIH1), and TIGIT- PE/Dazzle594 (A15153G). PBMCs were washed twice with the staining buffer and stained 30 min in the dark at 4°C with fixable viability dye 520. Finally, PBMCs were washed twice in the same staining buffer and analyzed on FACS BD LSR Fortessa. Red: One day before atezolizumab initiation. Blue: Five weeks after atezolizumab initiation. Green: Healthy control subject. FMO, fluorescence minus one. [file Image_2.tiff]
